# Knowledge, Attitudes, and Oral Health Practices of School Children in Davangere

**DOI:** 10.5005/jp-journals-10005-1358

**Published:** 2016-06-15

**Authors:** Satish Vishwanathaiah

**Affiliations:** Associate Professor, Department of Pedodontics, College of Dentistry, Jazan, Jazan University, Kingdom of Saudi Arabia

**Keywords:** Attitude, Knowledge, Oral hygiene, Practice, School children.

## Abstract

**Aims:** The aim of this study was to assess the knowledge, attitude, and behavior of school children towards oral health.

**Settings and design:** Descriptive study.

**Materials and methods:** School children (n=700) aged between 10 to 14 years in a Davangere school were recruited into this study. The subjects completed a questionnaire that aimed to evaluate young school children’s behavior, knowledge, and perception of their oral health and dental treatment.

**Statistical analysis:** The results were statistically analysed and percentage was calculated.

**Results and conclusion:** The participant oral hygiene habits (such as tooth brushing) were found to be irregular, and parent role in the oral hygiene habits of their children was limited. The study population showed higher awareness of caries than periodontal conditions. The children in this study also recognized the importance of oral health. The results of this study indicate that Comprehensive oral health educational programs for both children and their parents are required to achieve this goal.

**How to cite this article:** Vishwanathaiah S. Knowledge, Attitudes, and Oral Health Practices of School Children in Davangere. Int J Clin Pediatr Dent 2016;9(2):172-176.

## INTRODUCTION

The American Dental Association recommends that to avoid oral diseases individual should brush and floss at least once a day and visit a dentist regularly.^1^ We all know that lack of oral hygiene practices leads to development of oral diseases. These practices, such as brushing, flossing, and periodic dental visit should be developed early in the childhood. Dental flossing and tooth brushing are the most commonly performed oral self-care behavior.^2^ About one-third of the sample reported flossing at least once a day. Three quarters of the population reported making periodic dental visits at least once a year.^3^ Most of the children (73-83%) in Sweden, Denmark, Germany, Austria, and Norway brushed their teeth twice a day. The use of the dental floss was rare. In general, flossing was less frequent among boys than among girls.^4^ Recent studies carried out in Michigan have found that, on an average, subjects reported brushing their teeth twice daily. Around 90% of the population brushed at least once a day. African-Americans are less likely than whites to brush and floss thoroughly and get regular dental checkup.^5^

So this study was done to assess the knowledge, attitude, and behavior of school children toward oral health in Davangere population.

## MATERIALS AND METHODS

A total of 700 school children in the age group of 10 to 14 years were included in this study. Permission from the concerned authorities was taken in prior. Before and after the survey procedure, all the student were subjected to Oral Hygiene Index - Simplified (OHI-S) and assessed. In order to assess knowledge, attitude, and practice regarding oral health, a questionnaire^6^ having 22 questions was prepared for the school children. With the permission from the dean of the school, dental health questionnaire was explained to the students. Later, the questionnaire was distributed among the children in the classrooms at the starting of the session and collected at the end of the session.

## RESULTS

The results were tabulated, percentage was calculated, and conclusions were drawn. The survey presented a comprehensive overview of the knowledge, attitudes, and practices of school children aged 10 to 14 years in a Davangere school.

Table 1 shows that 70.42% of the children showed poor oral hygiene prior to the study. After the survey, OHI-S analysis indicated that 62% of the children improved and showed good oral hygiene.

**Table 1:** **Table 1:** Simplified Oral Hygiene Index

			*Before*			
		*Total*			*%*	
Good		15			2.14	
Fair		183			26.14	
Poor		493			70.42	
			*After*			
		*Total*			%	
Good		438			62.57	
Fair		193			27.57	
Poor		69			9.85	

In this part of the survey, (Table 2) knowledge toward oral hygiene was measured. All children demonstrated that oral health had significant role in general health.

According to this, 46.80% of children said that decay is caused due to irregular brushing, whereas 9.2% children accepted that irregular tooth brushing causes gum diseases, and 8% of children concluded that irregular tooth brushing causes bad breath. Around 17.20% of children informed that all the factors were responsible for irregular tooth brushing.

**Table 2:** **Table 2:** Knowledge toward oral hygiene by school children in a Davangere school

*K1.* *Has oral health got any role on general health?*							
				*Total*		*%*	
		a. Yes		700		100	
		b. No		–		–	
		c. Don’t know		–		–	
*K2.* *What does irregular tooth brushing cause?*							
				*Total*		*%*	
		a. Decay		327		46.80	
		b. Gum disease		65		9.20	
		c. Bad breath		56		8	
		d. Stains on teeth		131		18.8	
		e. Nothing		–		–	
		f. All of the above		121		17.20	
*K3.* *Why do we get dental problems?*							
				*Total*		*%*	
		a. Eating sweets and ice creams		448		64	
		b. Not brushing properly		129		18.42	
		c. Not rinsing the mouth		6		0.85	
		d. Not regularly visiting a dentist		14		2	
		e. All of the above		103		14.71	
*K4.* *How can we prevent dental problems?*							
				*Total*		*%*	
		a. Avoiding sweets and sticky foods		338		48.28	
		b. Brushing properly		57		8.14	
		c. Mouth rinsing after meals		3		0.43	
		d. Regularly visiting a dentist		19		2.72	
		e. All of the above		283		40.43	
*K5.* *Do you know that clean mouth can prevent tooth decay?*							
				*Total*		*%*	
		a. Yes		674		96.29	
		b. No		26		3.71	
*K6.* *Do you know that a dentist can clean and polish your teeth?*							
				*Total*		*%*	
		a. Yes		700		100	
		b. No		–		–	
*K7.* *What can be prevented by regular cleaning of mouth?*							
				*Total*		*%*	
		a. Bleeding from gums		50		7.14	
		b. Loosening of gums		44		6.29	
		c. Loss of teeth		3		0.43	
		d. Bad smell		405		57.86	
		e. All of the above		198		28.28	

**Table 3:** **Table 3:** Attitude toward oral hygiene by school children in a Davangere school

*A1* . *Do you think maintaining healthy mouth is an individualresponsibility?*							
				*Total*		*%*	
		a. Yes		696		99.43	
		b. No		4		0.57	
*A2. Have you visited a dentist before?*							
				*Total*		*%*	
		a. Yes		328		46.86	
		b. No		372		53.14	
*A3. If yes, then for what reason?*							
				*Total*		*%*	
		a. Decay		156		22.29	
		b. Pain		68		9.71	
		c. Filling		23		3.29	
		d. Extraction		19		2.71	
		e. Any others specify		62		8.86	
A4. *Do you think it is required to visit a dentist periodically tomaintain the oral health?*							
				*Total*		*%*	
		a. Yes		683		97.57	
		b. No		17		2.43	

About 18.42% of children complained that dental problem occurs due to improper brushing. Around 64% attributed eating fruits and ice cream as causative factors, and 14.7% of children accepted that all factors contribute for development of a dental problem. Only 2% children said that dental problems occur because of not visiting the dentist regularly.

About 8.14% children informed that by proper brushing method, dental problem can be prevented, and 48.28% children accepted the fact that by avoiding sweets and sticky food dental problems can be resolved. Only 0.43% of children said that by rinsing mouth after meals dental problems can be reduced.

Around 96.29% of children accepted that cleaning can prevent tooth decay, and 3.71% of children contradicted it. About 57.86% children said that bad smell can be controlled by regular cleaning of mouth, and 7.14 and 6.29% children accepted that regular cleaning of mouth control bleeding gums and loosening of gums respectively. Around 28.28% of children accepted that regular cleaning of mouth can prevent all the causative factors. All children knew that dentist can clean and polish their teeth.

In this section of the survey, (Table 3) attitude of the school children was measured.

About 99.43% of children knew that maintenance of healthy mouth is each individual responsibility. Around 46.86% of children had visited the dentist, and 53.14% of children hadn’t visited the dentist before. It was noted that 22.29% of children visited dentist for decay reason, 9.71% for reason of pain, 3.29% constituted for filling, and 2.71% for extraction. Around 8.86% of children visited for other reasons like cleaning (oral prophylaxis) and a few for orthodontic consultation. Around 97.57% of children knew that periodic dental visit is required for maintaining good oral health. Only 2.43% children were unaware of it.

**Table 4:** **Table 4:** Practice toward oral hygiene by school children in a Davangere school

*P1. How do you clean your teeth?*							
				*Total*		*%*	
		a. Toothbrush and toothpaste		700		100	
		b. Toothbrush and tooth powder		–		–	
		c. Finger and tooth powder		–		–	
		d. Neem sticks		–		–	
		e. Any other specify		–		–	
*P2. How often you clean your teeth?*							
				*Total*		*%*	
		a. Once daily		487		69.57	
		b. Twice daily		213		30.43	
		c. More than twice daily		–		–	
		d. After every meal		–		–	
*P3. How do you brush your teeth?*							
				*Total*		*%*	
		a. Use horizontal strokes		208		29.71	
		b. Use vertical strokes		48		6.86	
		c. Both in horizontal and vertical directions		350		50	
		d. Circular strokes		94		13.43	
*P4. How often you change your brush?*							
				*Total*		*%*	
		a. Once in 3 months		216		30.86	
		b. Once in 6 months		304		43.43	
		c. Yearly once		4		0.57	
		d. When bristles get frayed up		155		22.14	
		e. Don’t know exactly		21		3	
P5. *What amount of paste you apply on your brush?*							
				*Total*		*%*	
		a. Full length of bristles		374		53.43	
		b. Half-length of bristles		249		35.57	
		c. Pea-sized amount		77		11	
*P6. Do you press the paste in between the bristles?*							
				*Total*		*%*	
		a. Yes		269		38.43	
		b. No		431		61.57	
*P7. Do you rinse your mouth after meals?*							
				*Total*		*%*	
		a. Yes		24		3.43	
		b. No		646		92.29	
		c. Sometimes		30		4.28	
*P8. Do you clean your tongue?*							
				*Total*		*%*	
		a. Yes		694		99.14	
		b. No		6		0.86	
*P9. How do you clean your tongue?*							
				*Total*		*%*	
		a. Tongue cleaner		553		79	
		b. Fingers		141		20.14	
		c. Toothbrush		–		–	
		d. Any others specify...		–		–	

In this section of survey, (Table 4) practice toward oral hygiene was measured. According to this all children used toothbrush and toothpaste for cleaning the oral cavity. Almost 69.57% of children brushed their teeth once daily and 30.43% children brushed twice. About 29.71% children brushed teeth with horizontal strokes, 6.86% children brushed with vertical strokes, and 50% of children brushed both in horizontal and vertical direction. Only 13.43% children used circular strokes for brushing.

Around 43.43% of children changed their toothbrush once in 6 months, and 30.86% children changed toothbrush every 3 months. Only 0.57% children changed toothbrush once a year, and 22.14% changed toothbrush when bristles frayed up. About 3% of children were unaware of the time of changing the toothbrush. Around 53.43% children applied toothpaste to full length of bristles, 35.57% applied to half-length of the bristles, and 11% of children applied pea-sized amount of toothpaste. Only 38.43% applied the paste in between the bristles where as 61.57% children didn’t apply the paste in between the bristles.

Around 92.29% children did not rinse the mouth after meals, 3.43% did rinse the mouth regularly, and 4.28% did rinse mouth sometimes. Again, 99.14% children did clean their tongue, and only 0.86% did not clean the tongue. About 79% of children did use the tongue cleaner for cleansing their tongue, and 20.14% of children used their finger to clean their tongue.

## DISCUSSION

Oral health is an integral component of general health. Childhood is the age where children develops reflexes to maintain general hygiene practices and attitude toward health. Children can be ruptured well for their general and oral health.^7^ It is the duty of parents, teachers as well as children themselves to know the importance of oral hygiene. We can target children for educating and motivating them for oral health maintenance and awareness. It is important to start oral health education in their regular curriculum at school level. This will help not only in creating awareness but also in development of correct oral health practices thereby to control orodental problems. In many countries, a large number of children and parents have limited knowledge on the causes and prevention of the most common oral diseases.^8^ This paper focuses on knowledge, attitude, and practices of school children toward the oral health. The school children showed very good response toward the study.

The OHI-S recorded showed that prior to the study, 70% of the children had poor oral hygiene. At the end of the study, there was a tremendous change in the children knowledge, attitude, and practice toward the oral health. The study showed that children attitude toward the oral hygiene should be guided properly by the parents and the guardians as well as the school dental health programs.^9^ To have an impact on attitude and practices, children may take more time, but in the long term it will have positive effects. Similar results are seen in the studies by Petersen and Torres.^10^

While assessing knowledge, children thought that irregular brushes cause only decay (46.80%), but they were less unaware of gum diseases and bad breath. This showed that around 64% of children commented that dental problems were caused due to eating ice creams only; however, other causes like improper brushing methods, not rinsing the mouth, and irregular visit to dentist were little cited by the children. And 48.28% children cited that dental problems can be prevented by avoiding sweets and sticky foods, whereas other prevention technique like proper brushing methods, rinsing mouth after meals, and regular dental visit were lesser known. These results indicated that improvement in knowledge toward learning proper brushing technique is needed. Interventions to increase the knowledge, regular visit to the dentist, subsequent use of flossing are essential and are in argument with other studies.^9^

While assessing the attitude of the school children, most of the children knew that the individual has prime responsibility toward maintaining healthy mouth and also that periodic dental visit is required to maintain oral health. Only 46.86% children had visited dentist, among which 22.29% visited for the reason of decay. Almost 53% children did not visit the dentist. This may be due to fear of dental setup,^11^ lack of toothache, or lack of parental encouragement.^12^ We also agree with the fact stated by Jalevik et al^13^ in 1999 that lack of parents’ regular dental attendance might be reflected in children dental attitude. The behavior displayed by parents might also be the cause of lack of attendance regarding visit to the dentist. Barker and Horton showed that delay in seeking dental care could be attributed to other factors like parental belief and practices, lack of economic resources, and accessibility of dental services. They also showed that parents play a vital role in influencing child’s oral health. Other factors, such as extraction and oral prophylaxis were not known to the patients. This concludes the fact that dental caries is one of the most common diseases in children. Other conditions like gingival and periodontal conditions, oral hygiene maintenance, orthodontic treatment was least considered. Lack of both parental and child oral health education might also explain these findings.^9^ So more educational program and importance of maintaining oral health to motivate children as well as the parents is required.

While assessing the oral hygiene practices of the children, all children used toothbrush and toothpaste as oral hygiene aids, as reported elsewhere.^14-17^ About 99.14% of children were aware of tongue cleansing methods out of which 79% children used tongue cleaner. Only 30.43% of children bushed twice daily pertaining to the fact that parental guidance toward oral health education is still lacking. Survey found that high percentage of children brushed their teeth once daily only. The same results were seen in the study done by Al-Omari MK et al 2006 in North Jordan.^12^ Lack of both parental and child oral health education might explain these findings.^18^ The other reasons may be due to the age of our children who were included in the survey that they try to achieve independence and start their attempts to build their own identity without family interference. Lack of both parental and child oral health education may explain this.^13^ Out of 700 school children, only 13.43% of children was aware of circular strokes used while brushing (Fones technique). More than 50% of children were not knowing about changing of the toothbrush at regular intervals, that is, once in 3 months. Only 53.43% children did apply toothpaste to length of bristles, and 38.43% children did press the toothpaste between the bristles. These factors correlate the fact that parental and school dental health guidelines were still lacking in the children.^18^ Regular school health program/education explaining these should be advised.

The oral health knowledge, attitude, and practice among the secondary school students are still below the satisfactory level. This awareness on importance of oral health should be motivated. As stated by Al-Omari MK et al^12^ we also accept that there is a need to decrease dependency on oral health personnel and encourage students to take responsibilities for their own oral health. As children spend their entire day in the school, schools are the advisable platform for educating, promoting oral health care. At this age, children are receptive to guidance and familiar with learning environment and culture. It is very important to target oral health education to the children since the lifestyle and hygiene practices once established at an early age can go a long way in spending rest of the life in a healthy way.^19^ It is important to include oral health education into school curriculum so that it is taken as a part of life. Countries like Australia and New Zealand have a very well-developed school oral health education program and have demonstrated decline in dental caries among school children in the last few decades.^20^

From our study we conclude:

- Oral health education programs has to be conducted in all the school in large scale, and even parents should also be a part of such educational programs.
- Parents’ education is a must and should be included to promote preventive oral care. It can be achieved if school authorities permit to educate them on parent teacher meeting.
- Teachers should be given education so that they can impart knowledge and importance on oral health and its maintenance.
- Oral preventive care should be included in school curriculum so that children and teachers know the importance of the same.
